# Effect of Anisotropy on the Resilient Behaviour of a Granular Material in Low Traffic Pavement

**DOI:** 10.3390/ma10121382

**Published:** 2017-12-03

**Authors:** Peng Jing, Hossein Nowamooz, Cyrille Chazallon

**Affiliations:** ICube, UMR7357, CNRS, Université de Strasbourg, INSA de Strasbourg, 24 Boulevard de la Victoire, 67084 Strasbourg CEDEX, France; hossein.nowamooz@insa-strasbourg.fr (H.N.); cyrille.chazallon@insa-strasbourg.fr (C.C.)

**Keywords:** granular material, repeated load triaxial tests, resilient deformation, anisotropy, finite element method

## Abstract

Granular materials are often used in pavement structures. The influence of anisotropy on the mechanical behaviour of granular materials is very important. The coupled effects of water content and fine content usually lead to more complex anisotropic behaviour. With a repeated load triaxial test (RLTT), it is possible to measure the anisotropic deformation behaviour of granular materials. This article initially presents an experimental study of the resilient repeated load response of a compacted clayey natural sand with three fine contents and different water contents. Based on anisotropic behaviour, the non-linear resilient model (Boyce model) is improved by the radial anisotropy coefficient *γ*_3_ instead of the axial anisotropy coefficient *γ*_1_. The results from both approaches (*γ*_1_ and *γ*_3_) are compared with the measured volumetric and deviatoric responses. These results confirm the capacity of the improved model to capture the general trend of the experiments. Finally, finite element calculations are performed with CAST3M in order to validate the improvement of the modified Boyce model (from *γ*_1_ to *γ*_3_). The modelling results indicate that the modified Boyce model with *γ*_3_ is more widely available in different water contents and different fine contents for this granular material. Besides, based on the results, the coupled effects of water content and fine content on the deflection of the structures can also be observed.

## 1. Introduction

Low traffic pavements with a thin bituminous surfacing, granular base, and sub-base layers represent approximately 60% of the road network in France. 

In these pavements, granular layers play an important role in the overall performance of the structure, especially in deformation behaviours, which are characterized by a long-term mechanical process and a short-term mechanical process. The short-term behaviour concerns the instantaneous deformation of the granular layers, as excited during the repeated traffic loading. The long-term behaviour concerns the accumulation of permanent deformations, which is not studied in this work.

The mechanical behaviour of granular materials is generally investigated with repeated load triaxial tests (RLTT). These tests are used to simulate the loading conditions to describe either resilient behaviour [1,2,3,4] or permanent deformation [5,6,7,8].

In pavements, the granular layers are compacted to achieve maximum dry density. During the compaction process, these layers become anisotropic due to the vertical loads applied. As a result, the granular layers become more rigid vertically than horizontally. 

Several studies [9,10,11,12,13,14,15,16,17] have been carried out to study anisotropy in granular materials:

Casagrande and Carillo [9] have made a distinction for granular materials between initial anisotropy and the anisotropy induced by applied stress. The latter was defined as the ratio between axial and radial deformation under isotropic loading [12]. The initial anisotropy is mainly due to the shape and disposition of the grains, and the geometrical arrangement. Karasahin and Dawson [17] stated that it was possible to measure the anisotropy of resilient deformation of unbound granular material with a repeated load triaxial test.

Oda and Sudoo [13] indicated that anisotropy induced by applied stress was mainly due to the development of plastic deformation. The plastic deformation, meantime, can be influenced by the coupled effects of water content and fine content or suction [18]. As a result, anisotropy induced in granular layers becomes more complex.

Besides, the granular materials usually exhibit a complex non-linear response under repeated loading. A reliable description of resilient behaviour using experimental methods with or without soil suction control is time-consuming, and needs sophisticated equipment as well as trained personnel [19]. Hence, in recent years, various elasticity models have been proposed to predict the resilient behaviour based on resilient modulus and Poisson’s ratio [20,21,22] or volumetric and shear stress–strain relationships [23,24]. A comparison of the most widely used elasticity models in pavement design can be found in the following Ref. [25]. 

Among these models, the Boyce model [23] presents the advantage of being simple (three parameters), and correctly simulating the effect of the stress on the resilient behaviour (dilatancy for high values of ∆*q*/∆*p*). As mentioned above, the anisotropy of the granular materials is increasingly being recognized as a property that must be modelled to adequately describe the pavement’s mechanical behaviour. Hence, Hornych et al. [24] improved the Boyce model to include the anisotropy, and the new model showed a good agreement between measured and modelling results. However, the effects of water content and fine content on the anisotropy were not considered in this improvement.

In this context, this paper initially presents the resilient behaviour of a clayey sand (Missillac sand) with different water contents and three fine contents through the RLTT results. The results allow us to improve the Boyce model by considering the effects of water content and fine content on the anisotropic behaviour of the granular materials. A finite element modelling will be additionally performed by taking into account the proposed approach to calculate the deflection of a low traffic pavement constituted of granular materials with three different fine contents at different water contents.

## 2. Experimental Program

### 2.1. Material Studied

The studied material is Missillac fine sand. It is an alluvium sand that comes from the quarry of Missillac in France. This soil is used as subgrade soil in low traffic pavements for full-scale pavement tests at IFSTTAR (Institut Français des Sciences et Technologies des Transports, de l’Aménagement et des Réseaux) in Nantes, France.

**Figure 1:**
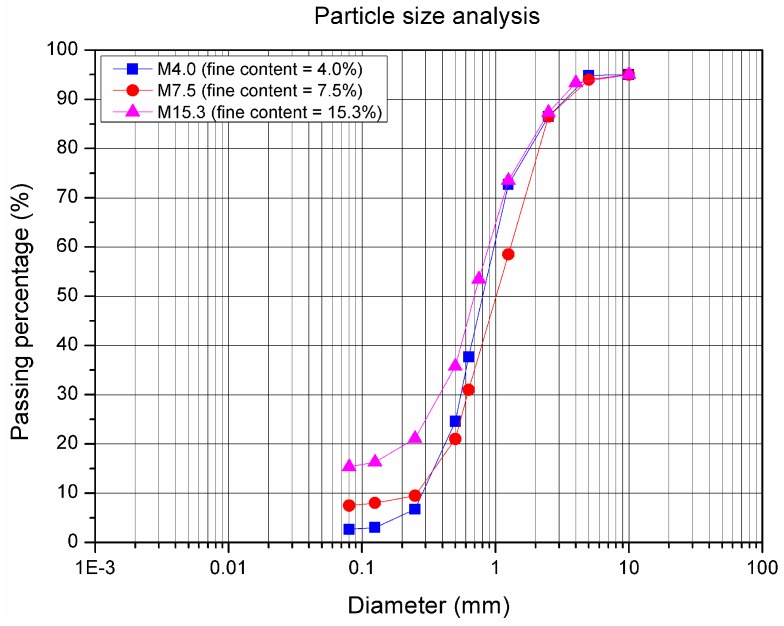
Particle size distribution curves of Missillac sand (M4.0, M7.5 and M15.3).

In this study, the natural samples have a maximum particle size of approximately 10 mm and contain three different fine contents (4.0%, 7.5%, and 15.3%, passing through the sieve 80 μm), which are named M4.0, M7.5, and M15.3. Figure 1 shows the particle size distribution for the three materials. Table 1 presents all of the characteristic parameters of these curves and the methylene blue values. Soils can also be classified based on these parameters [26,27], as reported in Table 1.

**Figure 2:**
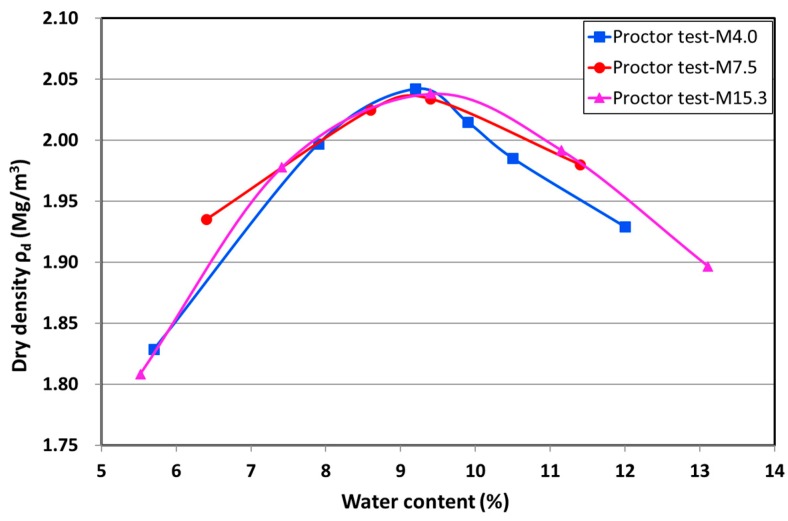
Standard Proctor compaction (M4.0, M7.5, and M15.3).

The standard Proctor compaction curves (Figure 2) show the optimum water content as between 9.1–9.5% and a maximum dry density of approximately 2.04 Mg/m^3^ for the three studied materials [28].

### 2.2. Repeated Load Triaxial Tests (RLTT)

Repeated load triaxial tests are performed on all three materials (M4.0, M7.5, and M15.3) with the water contents ranging between 7% and 11%, as shown in Table 2. The samples are compacted to the same dry density of 2 ± 0.06 Mg/m^3^ with two different methods (vibrocompression method and vibrating hammer method), as shown in Table 2. The size of the sample is large enough to avoid the particle size effect: it has a diameter of 150 mm/160 mm, and a height of 285 mm/320 mm (Table 2).

In this study, the RLTTs are conducted with a variable confining pressure (VCP), where both the axial load and the confining pressure are cycled in phase [29]. As a result, the sample under the cyclic stress (confining pressure *σ*_3_ and axial stress *σ*_1_) can be used to simulate the pavement solicitation and measure the vertical strain (*ε*_1_) and the radial strain (*ε*_3_). To determine the resilient behaviour of the materials, the sample is first subjected to a conditioning phase that consists of applying 10^4^ loading cycles to stabilize the permanent deformation:

- For the soil M4.0 and M15.3, the cyclic stresses applied during the conditioning were (∆*p*, ∆*q*) = (23.33 kPa, 70 kPa) from an initial stress state of (*p_0_*, *q_0_*) = (10 kPa, 0 kPa) at the frequency of 0.5 Hz;
- For the soil M7.5, the cyclic stresses applied during the conditioning were (∆*p*, ∆*q*) = (40 kPa, 80 kPa) from an initial stress state of (*p*_0_, *q*_0_) = (10 kPa, 5 kPa) at the frequency of 1 Hz. (The different loading method for M7.5 is used because this material is tested in Nantes with another machine).

*p* is the mean normal stress and *q* is the deviatoric stress. For a triaxial test, the *p* and *q* are defined as:

(1) $$p=\frac{\sigma_{1}+2\sigma_{3}}{3}$$

(2) $$q=\sigma_{1}-\sigma_{3}$$

where *σ*_1_ and *σ*_3_ are the major and minor principal stresses, respectively.

In this context, the permanent axial deformation achieves the equilibrium state after 10^4^ loading cycles. As a result, the study of resilient behaviour can be continued: after the conditioning phase, the samples (M4.0, M7.5, and M15.3) are subjected to a series of loads with different stress paths of ∆*q*/∆*p* = 0; 0.5; 1; 2; 3 [29,30], as shown in Table 3. Each stress path contains 100 loading and unloading cycles. The last cycle in each load sequence is used to determine the resilient behaviour. The soil resilient behaviour is generally presented by the volumetric strain (*ε_v_*) and the shear or deviatoric strain (*ε_q_*) varying with the mean normal stress (*p*). These deformations can be related to the axial strain (*ε*_1_) and the radial strain (*ε*_3_):

(3) $$\varepsilon_{v}=\varepsilon_{1}+2\varepsilon_{3}$$

(4) $$\varepsilon_{q}=\frac{2\left(\varepsilon_{1}-\varepsilon_{3}\right)}{3}$$

### 2.3. Cyclic Resilient Behaviour and Anisotropy

#### 2.3.1. Resilient Volumetric and Deviatoric Deformation

**Figure 3:**
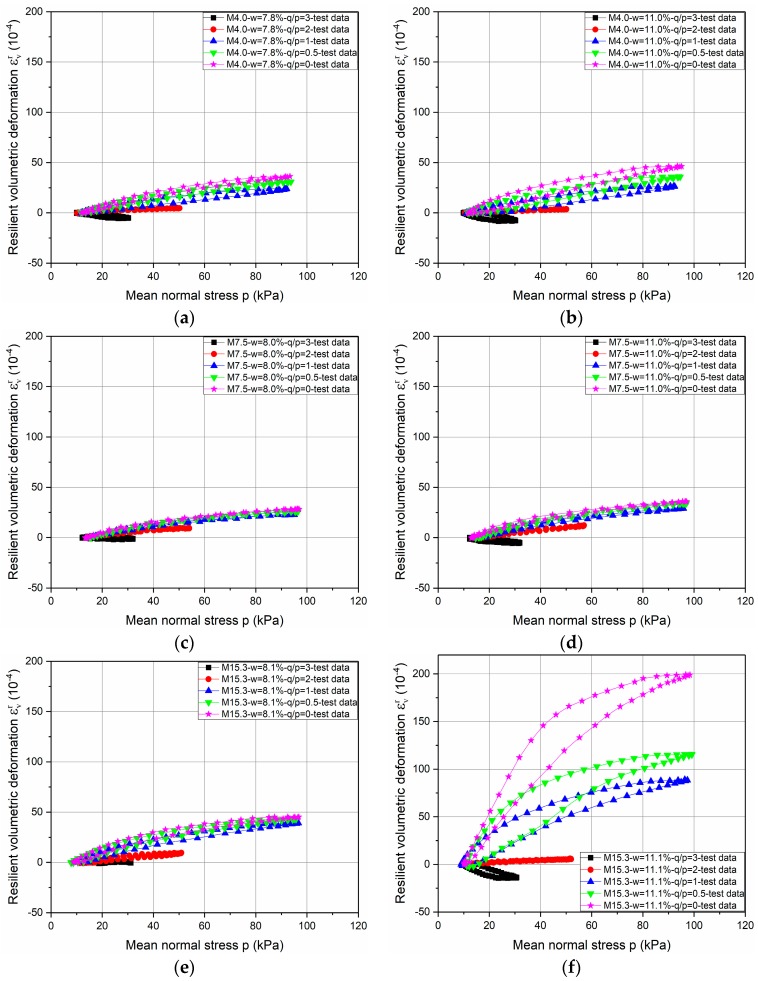
Evolution of resilient volumetric deformation *ε_v_^r^* for three different Missillac sands—M4.0, M7.5, and M15.3—with water contents of *w* = 8% and *w* = 11%. (**a**) M4.0-*w* = 7.8%; (**b**) M4.0-*w* = 11.0%; (**c**) M7.5-*w* = 8.0%; (**d**) M7.5-*w* = 11.0%; (**e**) M15.3-*w* = 8.1%; (**f**) M15.3-*w* = 11.1%.

**Figure 4:**
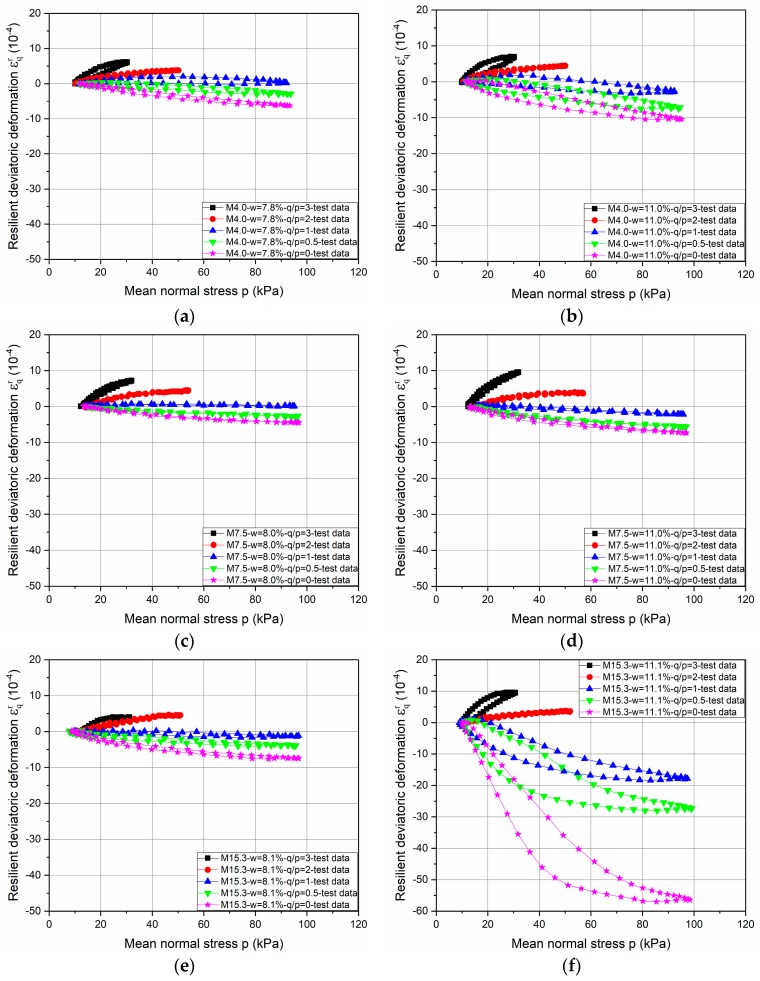
Evolution of resilient deviatoric deformation *ε_q_^r^* for three Missillac sands—M4.0, M7.5, and M15.3—with water contents of *w* = 8% and *w* = 11%. (**a**) M4.0-*w* = 7.8%; (**b**) M4.0-*w* = 11.0%; (**c**) M7.5-*w* = 8.0%; (**d**) M7.5-*w* = 11.0%; (**e**) M15.3-*w* = 8.1%; (**f**) M15.3-*w* = 11.1%.

Figure 3 and Figure 4 present respectively the resilient volumetric deformation *ε_v_^r^* and the resilient deviatoric deformation *ε_q_^r^* that are obtained for the different stress paths for the tests with water contents of 8% and 11% (approximately) for three different Missillac sands (M4.0, M7.5, and M15.3).

Based on the test results, it can be stated that the resilient behaviour of the soil is obviously non-linear, and depends on the mean stress *p* and the stress path ∆*q*/∆*p*. The coupled effects of water content and fine content on resilient behaviour are evident: when the water content is higher, the *ε_v_^r^* and the *ε_q_^r^* are then higher in each stress path for each material. However, the effect of fine content on resilient deformation is related to the soil saturation state. Besides, large increments of the values of *ε_v_^r^* and *ε_q_^r^* and open loops can be observed for M15.3 samples at water contents of 11.1% in the stress paths of ∆*q*/∆*p* = 0; 0.5; and 1. (The same phenomenon is obtained in M15.3 samples at water contents of 10.2%, 11.0%, and 11.3%).

#### 2.3.2. Anisotropy

**Figure 5:**
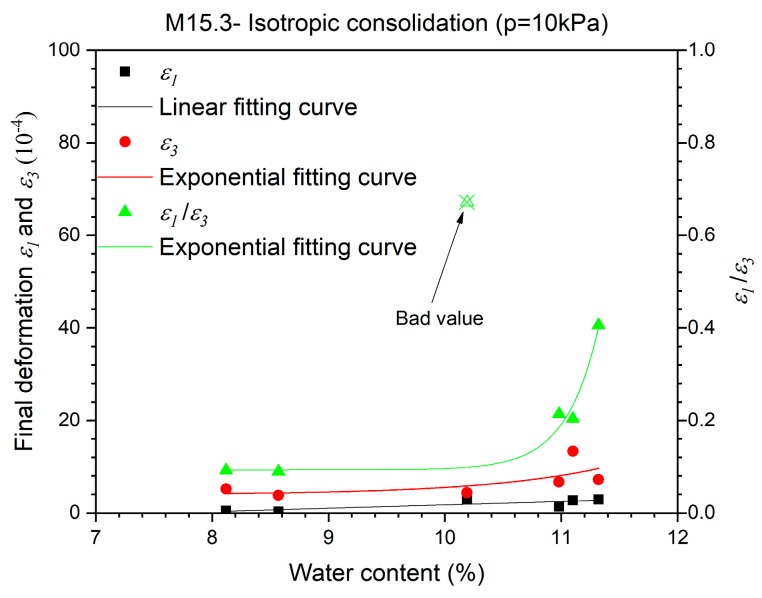
Final deformations *ε*_1_ and *ε*_3_ for Missillac sand M15.3 in an isotropic consolidation phase before conditioning.

Figure 5 presents the final deformations *ε*_1_ and *ε*_3_ of M15.3 samples with different water contents after an isotropic consolidation phase (∆*q*/∆*p* = 0, *p* = 10 kPa) before conditioning. It can be stated that there is an inherent anisotropy in these samples caused by compaction. The difference between *ε*_1_ and *ε*_3_ obviously decreases as water content for the samples increases, with a high degree of saturation (*w* >10.5%). The results of M4.0 and M7.5 are lost in the tests.

**Figure 6:**
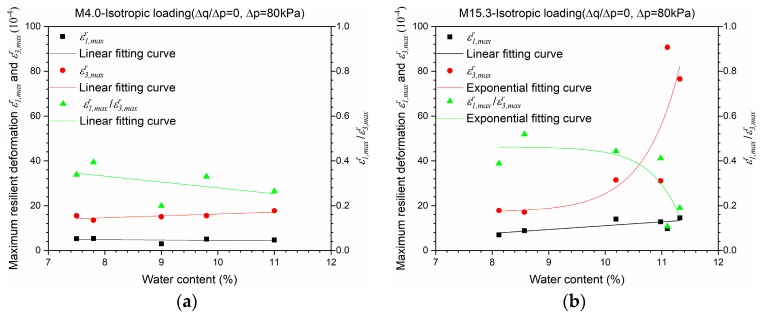
Maximum resilient deformations *ε*_1*,max*_*^r^* and *ε*_3*,max*_*^r^* for Missillac sands M4.0 and M15.3 in the isotropic loading stress path (∆*q*/∆*p* = 0) for the resilient test phase. (**a**) M4.0; (**b**) M15.3.

Figure 6 shows the maximum resilient deformations *ε*_1*,max*_*^r^* and *ε*_3*,max*_*^r^* for Missillac sand M4.0 and M15.3 in the isotropic loading stress path (∆*q*/∆*p* = 0, ∆*p* = 80 kPa) for the resilient phase. The anisotropy induced can be also observed obviously: the difference between *ε*_1*,max*_*^r^* and *ε*_3*,max*_*^r^* increases with water content for both materials, especially for M15.3.

Comparing the ratio value of *ε*_1_ to *ε*_3_ in Figure 5, and the ratio value of *ε*_1*,max*_*^r^* to *ε*_3*,max*_*^r^* in Figure 6b, it can be stated that the anisotropic behaviour of the granular material is very complicated and is strongly influenced by the stress history and the stress level applied. In the meantime, the comparison of the ratio value of *ε*_1*,max*_*^r^* to *ε*_3*,max*_*^r^* within Figure 6a,b shows that the anisotropic behaviour of M15.3 increases faster than M4.0 with an increase of water content from 7% to 11%.

Based on these results, it can be stated that the obvious inherent and developing anisotropic behaviour during loading phases shouldn’t be ignored.

## 3. Modelling

In this section, the modified Boyce model based on anisotropy coefficients *γ*_1_ and *γ*_3_ will be compared in order to describe the resilient behaviour of Missillac sand.

### 3.1. Modified Boyce Model

Boyce [23] proposed an isotropic non-linear model for predicting the resilient volumetric deformation and resilient deviatoric deformation of granular materials, which was expressed as:

(5) $$\varepsilon_{v}^{r}=\frac{1}{K_{a}}p^{n}(1-\beta \frac{q^{2}}{p^{2}})=\frac{1}{K_{a}}p^{n}\left[1+\frac{(n-1)\cdot K_{a}}{6G_{a}}{\left(\frac{q}{p}\right)}^{2}\right]$$

(6) $$\varepsilon_{q}^{r}=\frac{1}{3G_{a}}p^{n}\frac{q}{p}$$

where *β = (*1 *− n)·K_a_/(*6*G_a_)*; *K_a_* and *G_a_* are the reference bulk and shear moduli, respectively; and *n* is the nonlinear coefficient.

Hornych et al. [24] introduced the anisotropic response of granular materials into the Boyce model through multiplying the axial stress by an anisotropy coefficient *γ*_1_. Thus, the new mean normal stress p* and the new deviator stress q* could be re-expressed as follows:

(7) $$p^{*}=\frac{\gamma_{1}\sigma_{1}+2\sigma_{3}}{3}$$

(8) $$q^{*}=\gamma_{1}\sigma_{1}-\sigma_{3}\;，\;0<\gamma_{1}<1$$

where *γ*_1_ is the axial anisotropy coefficient.

Then, the potential function can be modified as:

(9) $$W=\frac{{\left[\frac{\gamma_{1}\sigma_{1}+2\sigma_{3}}{3}\right]}^{n+1}}{p_{a}{}^{n-1}}\left[\frac{1}{(n+1)K_{a}}+\frac{1}{6G_{a}}{\left(\frac{\gamma_{1}\sigma_{1}-\sigma_{3}}{\frac{\gamma_{1}\sigma_{1}+2\sigma_{3}}{3}}\right)}^{2}\right]$$

The new equations of resilient volumetric and deviatoric deformation could be proposed by taking the derivative of the potential function as:

(10) $$\varepsilon_{v}^{r}=\frac{p^{*}{}^{n}}{p_{a}{}^{n-1}}\left[\frac{\gamma_{1}+2}{3K_{a}}+\frac{n-1}{18G_{a}}\left(\gamma_{1}+2\right)\cdot {\left(\frac{q^{*}}{p^{*}}\right)}^{2}+\frac{\gamma_{1}-1}{3G_{a}}\cdot \frac{q^{*}}{p^{*}}\right]$$

(11) $$\varepsilon_{q}^{r}=\frac{2}{3}\cdot \frac{p^{*}{}^{n}}{p_{a}{}^{n-1}}\left[\frac{\gamma_{1}-1}{3K_{a}}+\frac{n-1}{18G_{a}}\left(\gamma_{1}-1\right).{\left(\frac{q^{*}}{p^{*}}\right)}^{2}+\frac{2\gamma_{1}+1}{6G_{a}}\cdot \frac{q^{*}}{p^{*}}\right]$$

In fact, the anisotropy coefficient *γ*_1_ is used to decrease the axial stress *σ*_1_ to fit with the radial stiffness in the elastic range. However, as presented in Figure 5 and Figure 6, the anisotropy behaviour changes sharply with water content for samples with a high fine content where the radial deformation *ε*_3_ is dominant, and the two-time relation for *ε*_3_ in Equation (3), should be also taken into account. As a result, we think that *γ*_1_ could be no longer applicable for these results, and the radial anisotropy coefficient *γ*_3_ is recommended with a broader scope. In other words, with the large difference between *ε*_1_ and *ε*_3_, it is supposed that there is no longer such a relationship where *γ*_3_ = 1/*γ*_1_, and the model for *γ*_3_ is more general.

Thus, p* and q* can be redefined as follows:

(12) $$p^{*}=\frac{\sigma_{1}+2\gamma_{3}\sigma_{3}}{3}$$

(13) $$q^{*}=\sigma_{1}-\gamma_{3}\sigma_{3}\;，\;\gamma_{3}>1$$

Similar to the above mentioned, the new resilient volumetric deformation *ε_v_^r^* and new resilient deviatoric deformation *ε_q_^r^* with anisotropy coefficient *γ*_3_ can be also proposed by taking the derivative of the potential function as:

(14) $$\varepsilon_{v}^{r}=\frac{p^{*}{}^{n}}{p_{a}{}^{n-1}}\left[\frac{1+2\gamma_{3}}{3K_{a}}+\frac{n-1}{18G_{a}}\left(1+2\gamma_{3}\right).{\left(\frac{q^{*}}{p^{*}}\right)}^{2}+\frac{1-\gamma_{3}}{3G_{a}}\cdot \frac{q^{*}}{p^{*}}\right]$$

(15) $$\varepsilon_{q}^{r}=\frac{2}{3}\cdot \frac{p^{*}{}^{n}}{p_{a}{}^{n-1}}\left[\frac{1-\gamma_{3}}{3K_{a}}+\frac{n-1}{18G_{a}}\left(1-\gamma_{3}\right)\cdot {\left(\frac{q^{*}}{p^{*}}\right)}^{2}+\frac{2+\gamma_{3}}{6G_{a}}\cdot \frac{q^{*}}{p^{*}}\right]$$

### 3.2. Modelling Based on Two Approaches (γ_1_ and γ_3_)

In this section, the modified Boyce model based on anisotropy coefficients *γ*_1_ and *γ*_3_ will be applied to describe the resilient behaviour of Missillac sand to demonstrate the improvement with *γ*_3_.

•Parameter optimization for stress paths of ∆*q/*∆*p* = 0.5, 1, 2 and 3

According to Equations (10) and (11) and Equations (14) and (15), the least square method is used to optimize the model parameters, *Ka*, *Ga*, *n,* and *γ*_1_ (*γ*_3_) for a given water content on stress paths of ∆*q/*∆*p* = 0.5, 1, 2 and 3.

•Parameter optimization for stress path of ∆*q/*∆*p* = 0

The resilient deformations of M15.3 samples at high water contents in the stress path of ∆*q*/∆*p* = 0 are obviously larger than other slopes (0.5, 1, 2 and 3), as shown in Figure 3f and Figure 4f. To obtain the good correlation coefficients for these samples, a different anisotropy coefficient *γ*_3_* is used for the stress path of Δ*q*/Δ*p* = 0 with the approach *γ*_3_ for all three materials. In the meantime, to keep things consistent, *γ*_1_* is also required for the stress path of ∆*q/*∆*p* = 0 with the approach *γ*_1_.

The model parameters, as well as the correlation coefficients, are presented in Table 4 for M4.0 samples, Table 5 for M7.5 samples, and Table 6 for M15.3 samples with different water contents.

Based on the results, it can be stated that almost the same correlation coefficient values (close or higher than 0.8) can be found for M4.0 samples with both methods (Table 4). The better correlation coefficient values (higher than 0.9) are observed and almost the same for M7.5 samples with both methods (Table 5). However, a significant improvement of correlation coefficients for M15.3 samples at the high water contents (10.2%, 11.0%, 11.1%, and 11.3%) can be observed from *γ*_1_(*γ*_1_*) to *γ*_3_(*γ*_3_*) (Table 6).

Besides, it can be noticed that the *γ*_1_ and *γ*_1_* values, as well as the *γ*_3_ and *γ*_3_* values, are almost constant or scatter slightly for M4.0 and M7.5 with all of the water contents (from 7.0% to 11.0%), as shown in Table 4 and Table 5. For the M15.3 samples, the *γ*_1_ and *γ*_1_* values, decrease and the *γ*_3_ and *γ*_3_* values obviously increase as the water content increases, as shown in Table 6.

**Figure 7:**
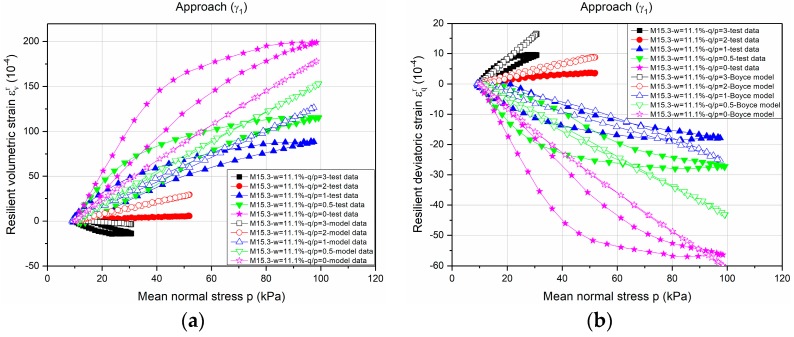
Modelling of *ε_v_^r^* and *ε_q_^r^* based on *γ*_1_ and *γ*_1_* for Missillac sand M15.3 at water content of *w* = 11.1%. (**a**) *ε_v_^r^*; and (**b**) *ε_q_^r^*.

**Figure 8:**
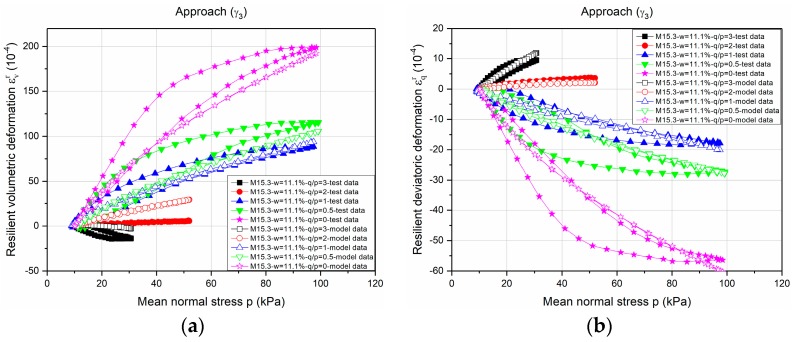
Modelling of *ε_v_^r^* and *ε_q_^r^* based on *γ*_3_ and *γ*_3_* for Missillac sand M15.3 at water content of *w* = 11.1%. (**a**) *ε_v_^r^*; and (**b**) *ε_q_^r^*.

Figure 7 and Figure 8 show the example of modelling the *ε_v_^r^* and *ε_q_^r^* values for Missillac sand M15.3 at water content of *w =* 11.1% based on *γ*_1_(*γ*_1_*) and *γ*_3_(*γ*_3_*), respectively. As described by the correlation coefficients above, an obvious improvement of the estimated results (both *ε_v_^r^* and *ε_q_^r^*) can be obtained.

Consequently, it can be stated that the radial anisotropy coefficient *γ*_3_, compared with the axial anisotropy coefficient *γ*_1_, can improve the prediction of the modified Boyce model for the granular materials with a high water content and a high fine content.

### 3.3. Relationship between γ_1_ and γ_3_ (γ_1_* and γ_3_*)

**Figure 9:**
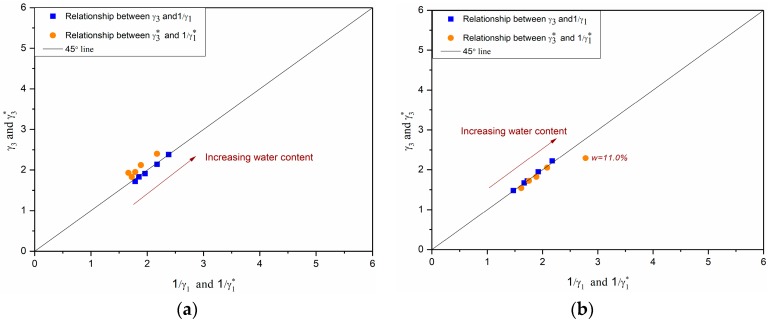

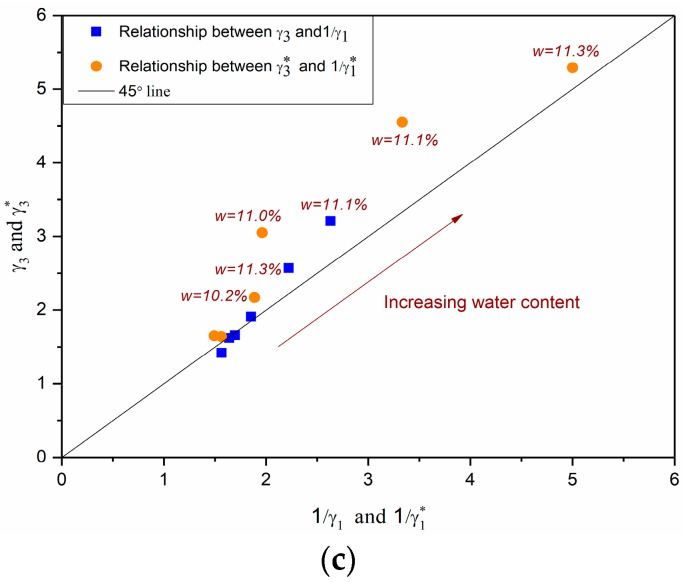
Relationships between *γ*_3_ and 1/*γ*_1_ and *γ*_3_* and 1/*γ*_1_* at water contents from 7% to 11%: (**a**) M4.0; (**b**) M7.5; (**c**) M15.3.

The values of *γ*_3_ and 1/*γ*_1_ and the values of *γ*_3_* and 1/*γ*_1_* are presented in Figure 9 for M4.0, M7.5, and M15.3 samples. Generally, the results show that the relations of *γ*_3_ = 1/ *γ*_1_ and *γ*_3_* = 1/*γ*_1_* can be obtained approximately in all of the samples of M4.0 and M7.5. For M15.3 samples, the relation of *γ*_3_ = 1/ *γ*_1_ fits well, except for samples at water contents of 11.1% and 11.3%. The relation of *γ*_3_* = 1/*γ*_1_* fits only with samples in two low water contents of 8.1% and 8.6%. 

The results confirm that the reciprocal relationship between the axial anisotropy coefficient and the radial anisotropy coefficient can change with the ratio of *ε*_1_*^r^* to *ε*_3_*^r^* after a threshold. The increase of this ratio or anisotropic behaviour is caused by the coupled effects of water content and fine content in this study. In fact, there are some other influences, such as the shape and disposition of the grains or the loading history, that can induce the anisotropic behaviour of granular materials. As a result, for more general behaviours for granular materials, it is necessary to take into account the variation of anisotropic behaviour as well.

### 3.4. Numerical Modelling of a Flexible Pavement Structure

The objective of this section is to compare the effects of two anisotropy coefficients, *γ*_1_ and *γ*_3_, on the deflection of a low traffic pavement structure. Finite elements modelling is performed with the finite elements code CAST3M.

**Figure 10:**
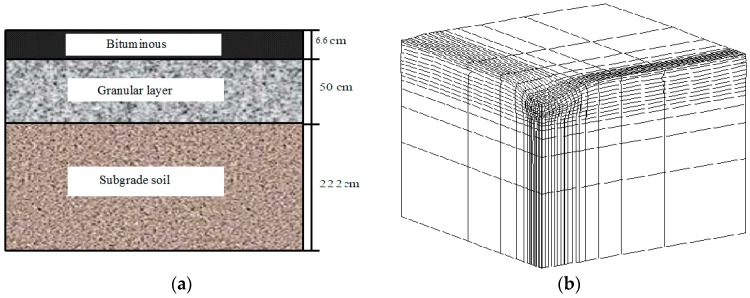
(**a**) Low-traffic pavement studied; (**b**) 3D finite element mesh for pavement simulation.

The pavement structure [33] consists of a 66mm of asphalt surface layer placed on 500 mm of unbound granular base over 2.22 m of clayey sand (Missillac) subgrade (Figure 10a). Due to the symmetry, the 3D calculation is carried out on a quarter of the structure (Figure 10b). The pavement is discretized into 2200 cubical elements with 20 nodes. The applied load is a 65 kN dual wheel load, corresponding to the standard axle load used in France for pavement design. The loading area is rectangular, with a length of 0.30 m and a width of 0.18 m. The gravity and lateral stresses are first applied to the pavement structure to establish the initial in situ stress states.

The following materials are used for the calculation:

- The asphalt concrete is used in the surface of pavement. The material behavior is assumed to be linear elastic. The Young elastic modulus (*E*) is equal to 6110 MPa, and its Poisson ratio is taken as equal to 0.35.
- For the subgrade layer, the Missillac sands (M4.0, M7.5, and M15.3) are used with different water contents (from 7% to 11%). The elastic behaviour is described by the modified Boyce model with anisotropy coefficients *γ*_1_ and *γ*_3_, respectively. The model parameters in Table 4, Table 5 and Table 6 are used for the calculation.
- For the unbound base layer, the material parameters of Missillac sand are used in the calculation instead of the parameters of unbound granular material in order to avoid the influence of dual variables on the comparison of *γ*_1_ and *γ*_3_.

**Figure 11:**
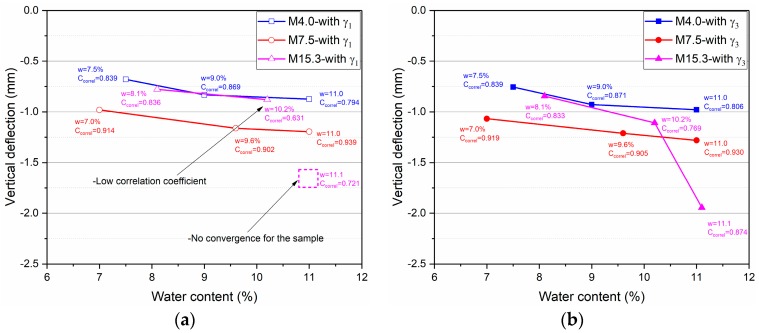
Calculation of the vertical deflection, (**a**) with *γ*_1_; (**b**) with *γ*_3_.

Figure 11 shows the deflections of the structures calculated by two different methods (*γ*_1_ and *γ*_3_) with three Missillac sands (M4.0, M7.5, and M15.3) at different water contents. It can be observed that the deflection values are identical with the two methods (*γ*_1_ and *γ*_3_) for all three materials at different water contents, except for the structures with M15.3 at water contents (10.2% and 11.1%, where *γ*_1_ ≠ 1/*γ*_3_). The difference of deflection between the two methods at a water content of 10.2% can be explained by the poor calculation with *γ*_1_ (Correl = 0.631). For the water content of 11.1%, the convergence cannot even reach with *γ*_1_. The simulation results show that the new proposed approach with anisotropy coefficient *γ*_3_ for the granular material has improved the modelling, especially for the case with large resilient deformation (at high water contents and high fine contents).

Besides, the coupled effect of water content and fine content on the deflection of the structures can be observed (Figure 11b): the deflections of the pavement will be obviously higher when the fine content increases from 4% to 7.5%. For the pavement with a high fine content of 15.3%, the matric suction is significantly higher in the unsaturated part because of more fine particles, which leads to a lower resilient deformation. As for the saturated part, the suction is very small, and more fine particles lead to decreasing the resistance of the resilient deformation because of the combination with water.

**Figure 12:**
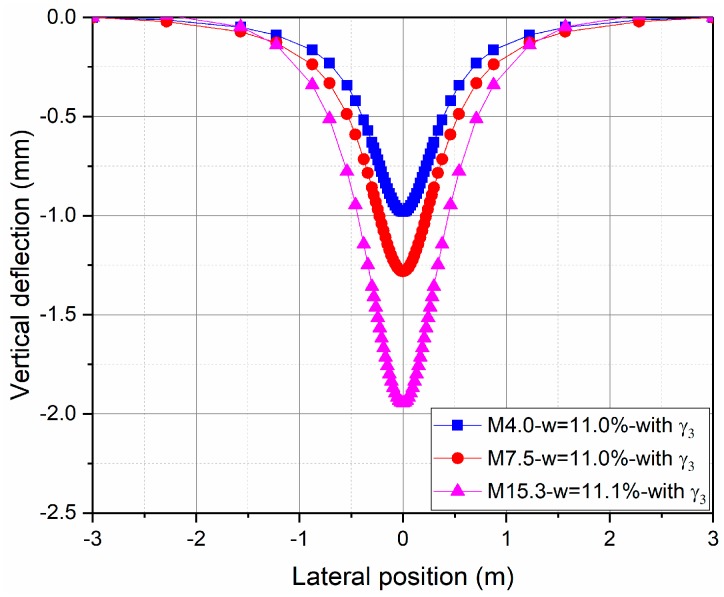
Calculation of the vertical deflection at *w* = 11% with *γ*_3_.

Figure 12 also shows the deflections of the structures with three fine contents at the water content of 11% using the new proposed approach (*γ*_3_ parameter). It can be seen that the evolution of deflection varies along the lateral position, and the effect of fine content is important.

## 4. Conclusions

This article initially presents the resilient behaviour of a natural compacted sand (Missillac sand), which is studied with RLTTs at different water contents and five stress paths varying from zero to three. The inherent anisotropy caused by compaction, and the anisotropy induced by loading stress, can be observed. The anisotropy is strongly influenced by the coupled effects of water content and fine content, which has a large increase as the water content increases (close to saturated state) for a high fine content sample.

Next, the radial anisotropy coefficient is suggested to replace the axial anisotropy coefficient in the non-linear model proposed by Boyce (1980), in order to determine the resilient behaviour of Missillac sand at three different fine contents and different water contents from 7% to 11%. The modelling of the results indicates that the radial anisotropy coefficient *γ*_3_, compared with the axial anisotropy coefficient *γ*_3_, show more general results, and thus obviously improves the accuracy of the modelling of this granular material with a high fine content close to saturated state. It also confirms the capacity of the improved model to capture the general trend of the experiments.

Finally, the finite elements modelling of a flexible pavement is performed with CAST3M to validate the improvement of the modified Boyce model (from *γ*_1_ to *γ*_3_). The modelling results present that the modified Boyce model with *γ*_3_ is more widely available in different water contents and different fine contents for this granular material. Besides, based on the results, the coupled effects of water content and fine content on the deflection of the structures can be observed apparently.

Consequently, the influence of the anisotropy on the resilient behaviour of granular materials has been seldom taken into account in modelling work. Furthermore, there is a strong link between the anisotropy behaviour and the coupled effects of water content and fine content. Understanding the complex mechanism of the anisotropic resilient deformation is helpful to propose more general resilient models or improve the existing models. This suggests that the number of tests used to predict the resilient behaviour for different moisture conditions or different fine contents can be reduced.

For the future work, this finding will be verified with different granular materials and even higher fine contents.

## Figures and Tables

**Table 1:** Characteristics of the studied materials.

Material	Dry Density (Mg/m^3^)	Fraction (%)				Particle Size					Blue Value	Classification	
		0/80 μm	0.08/0.4 mm	0.4/2 mm	2/5 mm	*d*60	*d*30	*d*10	*Cu*	*Cc*			
												NF	USCS
M4.0	2.00	4	10	76	5	0.95	0.55	0.30	3.17	1.06	---	B2	SP
M7.5	2.00	7.5	6.5	76	5	1.40	0.60	0.25	5.60	1.03	0.56	B2	SP-SC
M15.3	2.00	15.3	14.7	55	10	0.85	0.40	---	8.50	1.88	0.85	B5	SC

**Table 2:** Characteristics of the samples.

Material	Water Content (%)	Dry Density (Mg/m^3^)	Cylindrical Sample	Method of Compaction	Standard
M4.0	7.5; 7.8; 9.0; 9.8; 11.0	2 ± 0.06	150 mm × 285 mm	Vibrating hammer	NF EN 13286-4 [31]
M7.5	7.0; 8.0; 9.6; 10.0; 11.0	2 ± 0.06	160 mm × 320 mm	Vibrocompression	NF P 98-230-1 [32]
M15.3	8.1; 8.6; 10.2; 11.0; 11.1; 11.3	2 ± 0.06	150 mm × 285 mm	Vibrating hammer	NF EN 13286-4 [31]

**Table 3:** Stress paths applied in the resilient test phase.

Material	∆*q*/∆*p*	∆*p* (kPa)	∆*q* (kPa)
M4.0; M7.5; M15.3	0	80	0
	0.5	80	40
	1	80	80
	2	40	80
	3	20	60

**Table 4:** Parameter optimization of the modified Boyce model for M4.0.

***w* (%)**	**Δ*q*/Δ*p***	**Parameters (Missillac Sand, M4.0) with** ***γ*_1_ and** *γ*_1_*					**Correl**
		***Ka***	***Ga***	***n***	***γ*_1_**	*γ*_1_* (*q*/*p* = 0)	
7.5	0; 0.5; 1; 2; 3	17.88	18.95	0.77	0.51	0.56	0.839
7.8	0; 0.5; 1; 2; 3	20.01	27.52	0.56	0.56	0.58	0.767
9.0	0; 0.5; 1; 2; 3	16.30	15.48	0.62	0.42	0.46	0.869
9.8	0; 0.5; 1; 2; 3	17.87	16.00	0.74	0.54	0.60	0.841
11.0	0; 0.5; 1; 2; 3	16.48	10.60	0.88	0.46	0.53	0.794
Average values		17.71	17.71	0.72	0.50	0.55	0.822
***w*** **(%)**	**Δ*q*/Δ*p***	**Parameters (Missillac Sand, M4.0) with** ***γ*_3_** **and** *γ*_3_*					**Correl**
		***Ka***	***Ga***	***n***	***γ*_3_**	*γ*_3_* **(*q*/*p* = 0)**	
7.5	0; 0.5; 1; 2; 3	51.38	69.74	0.66	1.91	1.95	0.839
7.8	0; 0.5; 1; 2; 3	44.87	64.84	0.59	1.72	1.83	0.802
9.0	0; 0.5; 1; 2; 3	58.67	68.92	0.53	2.38	2.40	0.871
9.8	0; 0.5; 1; 2; 3	47.46	52.80	0.63	1.83	1.93	0.843
11.0	0; 0.5; 1; 2; 3	59.54	51.77	0.73	2.14	2.12	0.806
Average values		52.38	61.61	0.63	2.00	2.05	0.832

**Table 5:** Parameter optimization of the modified Boyce model for M7.5.

***w* (%)**	**Δ*q*/Δ*p***	**Parameters (Missillac Sand, M7.5) with** ***γ*_1_** **and** *γ*_1_*					**Correl**
		***Ka***	***Ga***	***n***	***γ*_1_**	*γ*_1_* (*q*/*p* = 0)	
7.0	0; 0.5; 1; 2; 3	8.49	31.91	0.23	0.52	0.48	0.914
8.0	0; 0.5; 1; 2; 3	11.97	35.47	0.27	0.60	0.57	0.931
9.6	0; 0.5; 1; 2; 3	9.29	30.15	0.22	0.58	0.53	0.902
10.0	0; 0.5; 1; 2; 3	12.77	45.25	0.22	0.68	0.62	0.942
11.0	0; 0.5; 1; 2; 3	4.15	27.10	0.11	0.46	0.36	0.939
Average values		9.33	33.98	0.21	0.57	0.51	0.926
***w* (%)**	**Δ*q*/Δ*p***	**Parameters (Missillac Sand, M7.5) with** ***γ*_3_** **and** *γ*_3_*					**Correl**
		***Ka***	***Ga***	***n***	***γ*_3_**	*γ*_3_* (*q*/*p* = 0)	
7.0	0; 0.5; 1; 2; 3	16.92	74.72	0.19	1.95	2.05	0.919
8.0	0; 0.5; 1; 2; 3	22.62	68.30	0.26	1.67	1.72	0.932
9.6	0; 0.5; 1; 2; 3	17.24	59.73	0.20	1.72	1.82	0.905
10.0	0; 0.5; 1; 2; 3	21.57	72.87	0.23	1.48	1.54	0.941
11.0	0; 0.5; 1; 2; 3	13.58	62.74	0.14	2.22	2.29	0.930
Average values		18.38	67.67	0.20	1.81	1.89	0.925

**Table 6:** Parameter optimization of the modified Boyce model for M15.3.

***w* (%)**	**Δ*q*/Δ*p***	**Parameters (Missillac Sand, M15.3) with** ***γ*_1_** **and** *γ*_1_*					**Correl**
		***Ka***	***Ga***	***n***	***γ*_1_**	*γ*_1_* (*q*/*p* = 0)	
8.1	0; 0.5; 1; 2; 3	14.46	22.01	0.74	0.59	0.64	0.836
8.6	0; 0.5; 1; 2; 3	15.78	25.78	0.66	0.61	0.67	0.838
10.2	0; 0.5; 1; 2; 3	10.90	13.51	0.99	0.64	0.53	0.631
11.0	0; 0.5; 1; 2; 3	11.03	10.13	0.91	0.54	0.51	0.645
11.1	0; 0.5; 1; 2; 3	4.90	2.95	1.00	0.38	0.30	0.721
11.3	0; 0.5; 1; 2; 3	7.86	3.03	1.17	0.45	0.20	0.606
Average values		10.82	12.90	0.91	0.54	0.48	0.713
***w* (%)**	**Δ*q*/Δ*p***	**Parameters (Missillac Sand, M15.3) with** ***γ*_3_** **and** *γ*_3_*					**Correl**
		***Ka***	***Ga***	***n***	***γ*_3_**	*γ*_3_* (*q*/*p* = 0)	
8.1	0; 0.5; 1; 2; 3	34.39	55.37	0.72	1.66	1.64	0.833
8.6	0; 0.5; 1; 2; 3	33.31	64.86	0.59	1.62	1.65	0.833
10.2	0; 0.5; 1; 2; 3	21.36	61.19	0.49	1.42	2.17	0.769
11.0	0; 0.5; 1; 2; 3	15.06	69.36	0.19	1.91	3.05	0.747
11.1	0; 0.5; 1; 2; 3	24.86	40.10	0.48	3.21	4.55	0.874
11.3	0; 0.5; 1; 2; 3	18.30	48.88	0.28	2.57	5.29	0.810
Average values		24.55	56.63	0.46	2.07	3.06	0.811

## References

[1] Caicedo B., Coronado O., Fleureau J.M., Correia A.G. (2009). Resilient Behaviour of Non Standard Unbound Granular Materials. Road Mater. Pavement Des..

[2] Nowamooz H., Chazallon C., Arsenie M.I., Hornych P., Masrouri F. (2011). Unsaturated Resilient Behavior of a Natural Compacted Sand. Comput. Geotech..

[3] Bilodeau J.P., Doré G. (2012). Water Sensitivity of Resilient Modulus of Compacted Unbound Granular Materials Used as Pavement Base. Int. J. Pavement Eng..

[4] Salour F., Erlingsson S. (2015). Resilient Modulus Modelling of Unsaturated Subgrade Soils: Laboratory Investigation of Silty Sand Subgrade. Road Mater. Pavement Des..

[5] Hornych P., Corte J.F., Paute J.L. (1993). Etude des Déformations Permanentes sous Chargements Répétés de Trois Graves Non Traitées. Bull. Liaison Lab. Ponts Chaussées.

[6] Werkmeister S., Dawson A.R., Wellner F. (2004). Pavement Design Model for Unbound Granular Materials. J. Transp. Eng..

[7] Duong T.V., Tang A.M., Cui Y.J., Trinh V.N., Dupla J.C., Calon N., Canou J., Robinet A. (2013). Effects of Fines and Water Contents on the Mechanical Behavior of Interlayer Soil in Ancient Railway Sub-structure. Soils Found..

[8] Salour F., Erlingsson S. (2017). Permanent Deformation Characteristics of Silty Sand Subgrades from Multistage RLT Tests. Int. J. Pavement Eng..

[9] Casagrande A., Carillo N. (1944). Shear Failure of Anisotropic Materials. Proc. Boston Soc. Civ. Eng..

[10] Gerrard C.M., Mulholland P. Stress Strain and Displacement Distributions in Cross-anisotropic and two Layer Isotropic Elastic Systems. Proceedings of the Australian Road Research Board (ARRB) Conference.

[11] Arthur J.R.K., Menzies B.K. (1972). Inherent Anisotropy in a Sand. Geotechnique.

[12] Biarez J., Hicher P.Y. Simplified hypotheses on mechanical properties equally applicable to sands and clays. Proceedings of the International Workshop on Constitutive Equations for Granular Non-Cohesive Soils.

[13] Oda M., Sudoo T. Fabric tensor showing anisotropy of granular soils and its application to soil plasticity in Powders and Grains. Proceedings of the 1st International Conference on Micromechanics of Granular Media.

[14] Hoque E. (1996). Elastic Deformation of Sands in Triaxial Tests. Ph.D. Thesis.

[15] Kohata Y., Tatsuoka F., Wang L., Jiang G.J., Hoque E., Kodaka T. (1997). Modelling the Non-linear Deformation Properties of Stiff Geomaterials. Geotechnique.

[16] Tutumluer E., Seyhan U. (1999). Laboratory Determination of Anisotropic Aggregate Resilient Moduli Using a New Innovative Test Device. Transp. Res. Rec..

[17] Karasahin M., Dawson A.R., Dawson A.R. (2000). Anisotropic characteristics of granular materials. Unbound Aggregates in Road Construction.

[18] Jing P., Nowamooz H., Chazallon C. (2018). Permanent Deformation Behaviour of a Granular Material Used in Low Traffic Pavements. Road Mater. Pavement Des..

[19] Han Z., Vanapalli S.K. (2016). State-of-the-Art: Prediction of Resilient Modulus of Unsaturated Subgrade Soils. Int. J. Geomech..

[20] Uzan J. (1985). Characterization of Granular Material. Transp. Res. Rec..

[21] Thom N.H., Brown S.F. The Effect of Grading and Density on the Mechanical Properties of a Crushed Dolomitic Limestone. Proceedings of the 14th Australian Road Research Board (ARRB) International Conference.

[22] Han Z., Vanapalli S.K. (2015). Model for Predicting Resilient Modulus of Unsaturated Subgrade Soil Using Soil-Water Characteristic Curve. Can. Geotech. J..

[23] Boyce H.R. A Non-linear Model for the Elastic Behaviour of Granular Materials under Repeated Loading. Proceedings of the International Symposium on Soils under Cyclic and Transient Loading.

[24] Hornych P., Kazai A., Piau J.M. Study of the Resilient Behavior of Unbound Granular Material. Proceedings of the 5th Conference on Bearing Capacity of Roads and Airfields.

[25] Laloui L., Charlier R., Chazallon C., Erlingsson S., Hornych P., Pavsic P., Srsen M., Dawson A. (2009). Water Influence on Mechanical Behaviour of Pavements: Constitutive Modelling. Water in Road Structures: Movement, Drainage and Effects.

[26] Association Française de Normalisation (AFNOR) (1992). Exécution des Terrassements—Classification des Matériaux Utilisables Dans la Construction des Remblais et des Couches de Forme D’infrastructures Routières.

[27] American Society for Testing and Materials (ASTM) (2011). Standard Practice for Classification of Soils for Engineering Purposes (Unified Soil Classification System).

[28] Association Française de Normalisation (AFNOR) (2014). Sols: Reconnaissance et Essais—Détermination des Références de Compactage d’un Matériau—Essai Proctor Normal. Essai Proctor Modifié.

[29] Association Française de Normalisation (AFNOR) (2004). Melanges Avec ou Sans Liant Hydraulique Essai Triaxial Sous Charge Cyclique Pour Melanges Sans Liant Hydraulique.

[30] American Association of State and Highway Transportation Officials (AASHTO) (2012). Standard Method of Test for Determining the Resilient Modulus of Soils and Aggregate Materials.

[31] Association Française de Normalisation (AFNOR) (2003). Mélanges Traités et Mélanges non Traités aux Liants hydrauliques. Méthodes L’essai Pour la Masse Volumique de Référence et la Teneur en Eau en Laboratoire—Marteau vibrant.

[32] Association Française de Normalisation (AFNOR) (1992). Préparation des Matériaux Traités aux Liants Hydrauliques ou non Traités. Fabrication des Eprouvettes par Vibrocompression.

[33] Chazallon C., Allou F., Hornych P., Mouhoubi S. (2009). Finite Elements Modelling of the Long-term Behaviour of a Full-scale Flexible Pavement with the Shakedown Theory. Int. J. Numer. Anal. Methods Geomech..

